# Manipulating XXY Planar Platform Positioning Accuracy by Computer Vision Based on Reinforcement Learning

**DOI:** 10.3390/s23063027

**Published:** 2023-03-10

**Authors:** Yi-Cheng Huang, Yung-Chun Chan

**Affiliations:** 1 Department of Mechanical Engineering, National Chung Hsing University, Taichung 40227, Taiwan; 2 Department of Mechatronics Engineering, National Changhua University of Education, Changhua City 50074, Taiwan; zxc86238623@gmail.com

**Keywords:** reinforcement learning, deep Q-learning network, computer vision

## Abstract

With the rise of Industry 4.0 and artificial intelligence, the demand for industrial automation and precise control has increased. Machine learning can reduce the cost of machine parameter tuning and improve high-precision positioning motion. In this study, a visual image recognition system was used to observe the displacement of an XXY planar platform. Ball-screw clearance, backlash, nonlinear frictional force, and other factors affect the accuracy and reproducibility of positioning. Therefore, the actual positioning error was determined by inputting images captured by a charge-coupled device camera into a reinforcement Q-learning algorithm. Time-differential learning and accumulated rewards were used to perform Q-value iteration to enable optimal platform positioning. A deep Q-network model was constructed and trained through reinforcement learning for effectively estimating the XXY platform’s positioning error and predicting the command compensation according to the error history. The constructed model was validated through simulations. The adopted methodology can be extended to other control applications based on the interaction between feedback measurement and artificial intelligence.

## 1. Introduction

Rapid developments in artificial intelligence (AI) have resulted in the creation of numerous AI applications for machining and automatic optical inspection systems. Many of these applications are aimed at improving the accuracy of positioning, the quality of machined parts, and the inspection of surface defects. The increasing demand for machine learning models has resulted in the development of customized software that strongly improves the performance and facilitates the development of machinery. Moreover, the demand for ultrafine workpieces inevitably leads to an increase in machining accuracy requirements. In particular, precision positioning platform systems must meet the requirements of smart manufacturing. For example, the XXY mask alignment stage system in [1] was developed to achieve precision positioning for dual charge-coupled device (CCD) cameras. An artificial neural network was developed to plan the motion of this system for nonlinear mapping from the desired position and orientation according to three motor control signal commands. Lee et al. [2] proposed a visual servo control and image alignment system for controlling the motion of a coplanar XXY platform. They conducted a kinematic analysis of the influence of setting error (between the workpiece center and the platform reference point) and used floating reference points to reduce the effect of this error for each alignment process. A long short-term memory (LSTM) network [3], which is a variant of a recurrent neural network, was first proposed in 1997. Because of its unique design, an LSTM network is often used to handle time-series data problems and to solve the vanishing gradient problem. Many studies have used LSTM networks to address various problems related to time-series data. The in-lab visual image recognition system introduced in [4] was constructed to record the displacement of the XXY platform. Feedback control from a CCD imaging system was used to reduce positioning errors. In this study, the positioning information of an XXY platform was acquired to construct an LSTM predictive model for a motion history time series. Platform displacement data were collected through image sensing, and the collected data were used to establish a training data set and testing data set for an LSTM network.

In [5], the use of response surface methodology was proposed for optimizing the parameters of an electric discharge machine for a machining process. The four factors (discharge duration, discharge current, capacitance, and discharge gap) and three levels of each factor proposed in [5] could effectively optimize output response variables. Most methods for determining the input parameters for any machining process are aimed at optimizing the output solutions on the basis of a known but constrained data set. However, searching for unknown parameters for a machine or machining process to improve solutions requires the application of AI. Selecting parameters or key features from an experimental data set for optimizing a process remains a challenging problem.

Machine learning has been developed to solve complex problems with various requirements by analyzing or predicting data. Reinforcement learning (RL) [6] was initially used to train game agents and achieved excellent results. In recent years, it has also been widely used in the fields of engineering, finance, and insurance as well as in self-driving car systems; stock market forecast analysis; and adjusting the servo parameters of machine tool controllers, which is relevant to this study.

The use of RL to automate the tuning process has provided promising results in recent years. RL was effective for identifying the most suitable parameters to achieve short processing time and small roundness in [7]. This method was used to adjust the proportional–integral–derivative (PID) controller parameters for a Cummins engine controller. The simulation results reveal that convergence can be achieved rapidly with respect to the rise time, settling time, maximum overshoot, and steady-state error. Therefore, the task can be completed effectively with fewer time steps. In [8], the control law of a variable-cycle engine in the cruise phase was constructed using a deep Q-learning network (DQN). A DQN algorithm was proposed to optimize the control law through simulation.

Thus, deep RL has become an effective method of solving problems for which an improved solution for a task is affected by the task conditions. RL optimizes agents (constructed by models) that can interact with their environment by optimizing agent behavior through environmental feedback signals. In [9], action–critic methods with a continuous action space for designing the feedback controller of a feed drive were implemented through RL. The positioning accuracy and response of the methods were superior to those of a conventional PID controller. A Distributed Proximal Policy Optimization (DPPO)-DQN was proposed to achieve obstacle avoidance for a working robot in [10]. The DQN and DPPO effectively performed obstacle-free navigation and solved the continuous action obstacle avoidance problem, respectively [10]. Recent studies have also discussed high-dimensional continuous action spaces. In [11], methods of inferring the DQN structure for high-dimensional continuous action spaces were studied using uncertainty estimation techniques.

In the present study, Q-learning was used with a temporal difference algorithm to achieve off-policy learning. To the best of our knowledge, this method has not been previously investigated. A precision-motion XXY platform was deployed in the experiments of this study. In-line feedback error from CCD camera images and offline positioning displacement from the dial indicator measurement data of the platform were input into the developed DQN model to train it to identify positioning error trends for each command. The DQN model could also predict and compensate for the position error of the platform. Such error compensation as well as time history prediction and analysis were realized through RL. The DQN could minimize consecutive ball-screw forward or backward rotations. The experimental results revealed that improved precision positioning methods do not need to track positioning states iteratively. The developed DQN model could also effectively compensate for the learning error.

## 2. RL Method

### 2.1. RL Fundamentals

**Figure 1:**
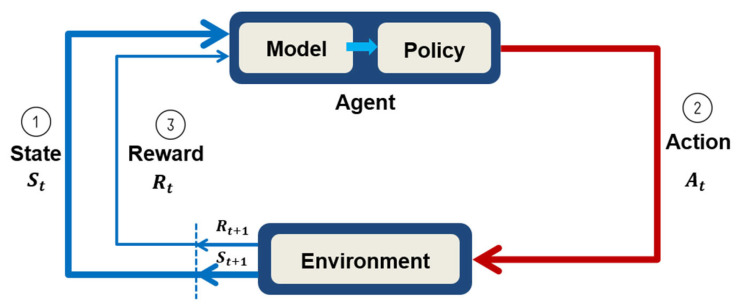
Markov decision process model [6].

RL can be considered a Markov decision process. The agent selects an action according to its policy by observing the environmental state and then obtains a reward. Presumably, discovering the best move for the next action should provide the greatest reward. As shown in Figure 1, the agent performs an action A_t at a certain time t, and the state in the environment changes from *S_t_* to *S*_(*t*+1)_.

According to its actions, the agent receives different rewards *R_t_*, and these rewards are used to evaluate the value of the action during the state transitions. Designing an appropriate reward mechanism and maximizing the learned rewards is the key challenge in RL. Finally, by updating the agent’s strategy after each feedback reward event, an optimal strategy can be obtained for performing the most valuable actions.

Policies are usually denoted by the symbol *π*. A value function that quantifies the agent’s performance in a certain state must be defined. This performance depends on the agent’s strategy, which is often represented as V(s). The value function is equal to the total expected reward received by the agent after starting in an initial state, and the best policy is that with the highest reward.

Agents interacting with an environment can perform model-based or model-free learning. In model-based learning, previously learned information is used to complete a task. By contrast, model-free learning relies on trial and error and involves selecting correct actions on the basis of previous experiences. Some basic RL equations are presented in Equations (1)–(8). In Equation (1), *P* is the state transition function, which describes the probability of state *s* transitioning to the next state *s′* when a specific action a is taken in *s* at time *t*. In Equation (2), *R* is the reward function, which describes the reward amount when *s* transitions to *s′* when *a* is taken in *s* at *t*. In Equation (3), *π* is the policy function, which describes the probability of a being executed when the agent observes the state *s* under policy *π*. In Equation (4), *G* is the sum of all rewards from *t* = 0 to the future, where *r_t_* is the reward obtained at time *t*. Moreover, γ is the discount factor, which is between 0 and 1. This term indicates an algorithm’s expectation for a future reward. If future events are prioritized, γ is close to 1. If γ is 0, future rewards are not considered, and only immediate rewards at *t* = 0 are valued. In Equation (5), *V* is the state-value function, *V^π^*(s) is the estimated sum of all future rewards, and *E* is the expected value for s under policy *π*. In Equation (6), V* finds a strategy *π* to maximize *V* for *s*. In Equation (7), *Q* is the action-value function, which is similar to the state-value function; however, *Q^π^(s, a)* is estimated by adopting strategy *π* for *s* and *a*. The term Q* in Equation (8) finds a strategy *π* that maximizes *Q* for *s* and *a*. The best state is often accompanied by the best action selected in that state. Therefore, by using the state-value and action-value functions, the state and the action can be calculated mathematically.

(1) $$P\left(s^{\prime },a\right)=P(S_{t+1}=s^{\prime }|S_{t}=s,A_{t}=a)$$

(2) $$P\left(s^{\prime },a\right)=R(S_{t+1}=s^{\prime }|S_{t}=s,A_{t}=a)$$

(3) $$\pi \left(s\right)=P\left(A_{t}=a|S_{t}=s\right)$$

(4) $$G=\overset{\infty }{\underset{t=0}{\sum }}\;\gamma^{t}r_{t}$$

(5) $$V^{\pi }\left(s\right)=E[G|s,\pi ]\;$$

(6) $$V^{*}\left(s\right)=max_{\pi }V^{\pi }\left(s\right)=max_{\pi }E[G|s,\pi ]$$

(7) $$Q^{\pi }\left(s,a\right)=E[G|s,a,\pi ]$$

(8) $$Q^{*}\left(s,a\right)=max_{\pi }Q^{\pi }\left(s,a\right)=max_{\pi }E[G|s,\pi ]$$

### 2.2. Q-Learning

**Figure 2:**
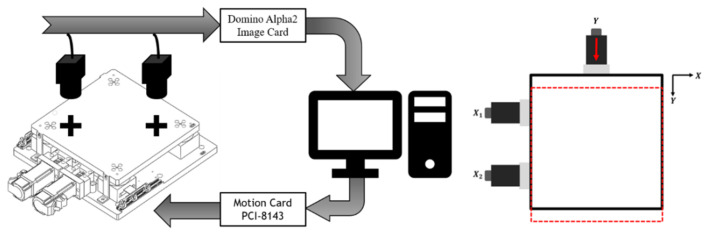
Configuration of the XXY stage system and illustration for the Y-axis motion in the experiment [4].

The main concept of the Q-learning algorithm is to train an agent, instead of humans or machines, to make decisions. This algorithm can solve problems through a value-based algorithm to produce a rational and objective decision. The main concept of the Q-learning algorithm [6] is illustrated in Figure 2. The Q-learning algorithm learns from the reward and punishment table, selects the next action, and then updates the Q-table. The values in the Q-table are constantly iteratively updated in accordance with the state transition score and action-value score until changes in the table values are extremely small and thus convergence has been achieved. At this point, the Q-table is no longer updated, and the best action is defined as the optimal decision for the value of a given state after training has been completed.

### 2.3. Deep Q-Network

The DQN is a classic algorithm for solving RL problems by using neural networks. This network was proposed by Google’s DeepMind team in 2015 and was published in the world-renowned journal *Nature*. In an Atari game, 30 of its 49 high-dimensional game outcomes surpassed the human level [12]. The DQN can expand the input data as a vector (with various values) or as image data. The output of this network is a corresponding action. The main function of the Q-learning and DQN algorithms can be found in [6].

## 3. XXY Visual Feedback Control System

### 3.1. XXY Platform Hardware

The experimental XXY platform contains three motors on the same plane and has a low center of gravity. The main advantage of the XXY stage is its smaller assembled error of stage composition compared with a traditional stacked stage. The coplanar XXY stage is popular for precision-motion applications in manufacturing, such as automatic optical inspection and lithography processes.

**Figure 3:**
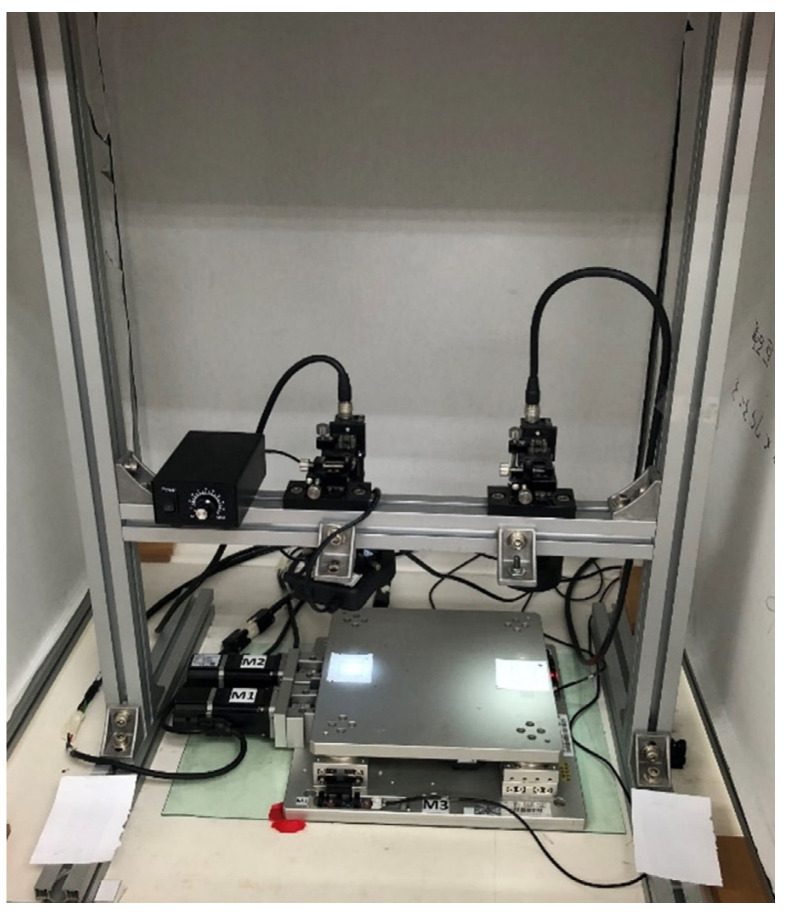
Photograph of the XXY experimental stage.

As shown in Figure 2, the coplanar XXY stage with two degrees of freedom (XXY-25-7, CHIUAN YAN Ltd., Changhua, Taiwan) [13] contains an image servo controller and two CCD cameras, which are mounted on the top of the system, as is the servo positioning sensor. A motion card (PCI-8143, ADLINK Technology Inc., Taoyuan City, Taiwan) controls the XXY stage [14], and ADLINK’s Domino Alpha2 image card is used for XXY stage image position feedback. A photograph of the XXY experimental stage is shown in Figure 3.

**Figure 4:**
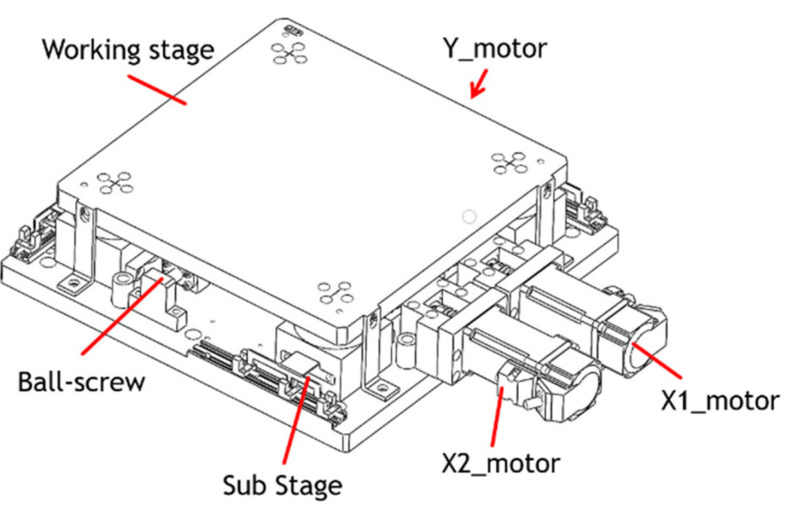
Coplanar XXY stage [13].

Conventional XYθ stages use a stacked design comprising an x-axis and a y-axis for translation and a θ-axis for rotation. However, the XYθ stage produces cumulative flatness errors because of its stacked assembly and large stage size. Therefore, a coplanar XXY stage was developed to reduce cumulative error and increase movement speed. Figure 4 displays the structure of a coplanar XXY stage, which is driven by an x_1_-axis servo motor, an x_2_-axis servo motor, and a y-axis servo motor. This stage has three degrees of freedom: the translation along the x-axis and y-axis. It achieves θ-axis rotation by actuating its _X1_-axis and _X2_-axis motors and halting its y-axis motor. The XXY stage can move up to ±5 mm with a maximal angle of ±2°.

### 3.2. Vision for the XXY Motion Stage

The purpose of the proposed method is to determine the position of the alignment symbol (a cross mark) through RL. The center-of-gravity method is used to obtain the target position in the image coordinate system. The coordinates of the stage are calculated on the basis of the coordinates of the cross mark. The DQN must locate the cross-mark position and compensate for the error corresponding to ball-screw stepping movements.

**Figure 5:**
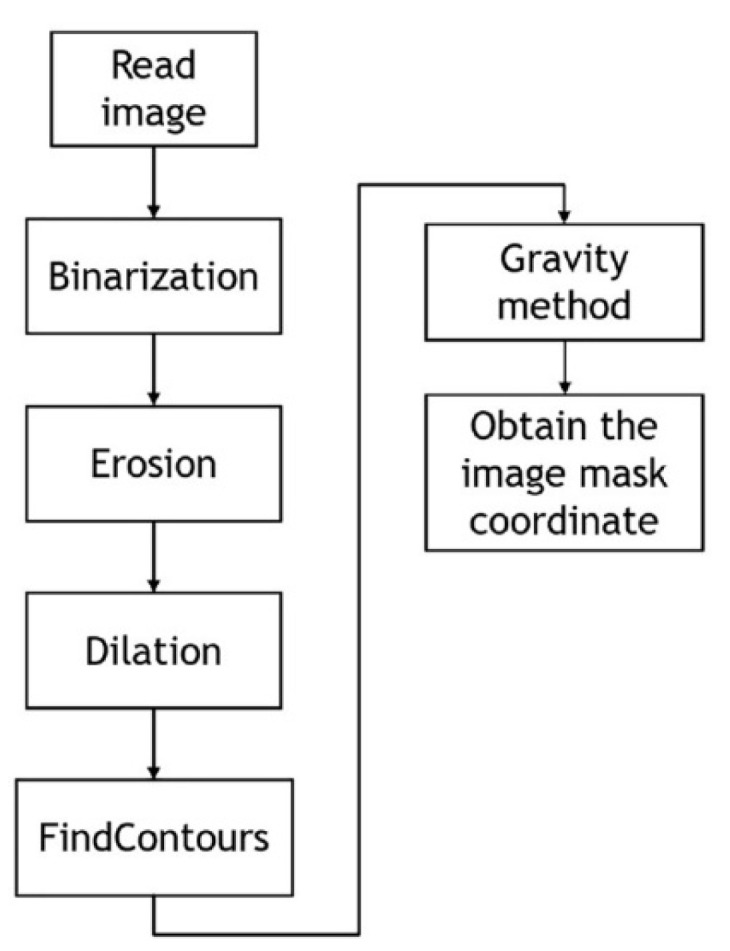
Image identification procedure [4].

**Figure 6:**
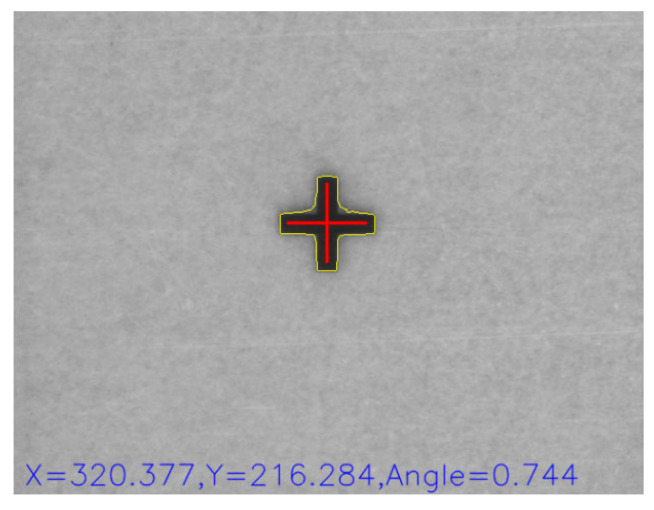
Cross-mask position obtained using the center-of-gravity method [4].

For the center-of-gravity method, grayscale images are acquired from multiple CCD cameras, and the image noise is removed using a filter. The binary threshold of the grayscale histograms is used to separate the two targets of the cross mark and the background. Expansion and subtraction are applied to remove the remaining noise, thereby enabling the optimal image to be produced through a morphological process. Subsequently, feature targets are identified using the findContours function of OpenCV (4.5.1). The center-of-gravity method can thus obtain the coordinates of the image center for the positioning mark. Figure 5 displays a flowchart of the image identification procedure, and Figure 6 displays the cross-mask position obtained by the center-of-gravity method.

### 3.3. XXY Stage Controller

**Figure 7:**
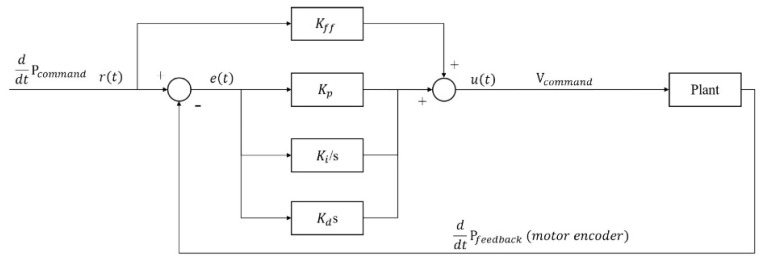
Architecture of PCI-8143 motion card controller [14].

The time-domain state of the PID controller for the XXY stage is expressed as follows:

(9) $$u\left(t\right)=K_{p}e\left(t\right)+K_{i}\int_{0}^{t}\;e\left(t\right)dt+K_{d}\frac{de\left(t\right)}{dt}+K_{ff}r\left(t\right)\;$$

where *r*(*t*), *e*(*t*), *u*(*t*), *K_p_*, *K_i_*, *K_d_*, and *K_ff_* represent the input command, system error, control variable, proportional gain, integral gain, derivative gain, and velocity feedforward gain, respectively. Figure 7 presents the architecture of the PCI-8143 motion card controller.

## 4. Experimental Methodology

### 4.1. Experimental Setup

The XXY platform was used for experiments of point-to-point y-axis movement for a reciprocating motion. For one-way motion, 10 displacement commands were performed in the same direction; each step command involved a movement of 100 μm. Each 10 forward displacement commands were followed by a return of backward motion. In the displacement process, a dial gauge measured the displacement generated by the actual platform movement. This displacement error was subtracted from the CCD feedback for semiclosed-loop sensing. The real closed-loop error compensation was predicted and learned by the constructed DQN. The ball nut preload loss or the backlash and frictional effect of the ball-screw drive system caused nonrepetitive positioning error; therefore, the RL model was first trained through offline learning to determine the platform offset error compensation. Simultaneously, the RL agent performed the CCD image assistance method to issue a correction for the desired command. Some platform point displacement errors are listed in Table 1.

### 4.2. State Design of the DQN Model

In this study, an XXY platform’s offset error data set was used to construct a DQN model. Because the collected data were time-series data, offset error might have accumulated because of the time-series problem, thereby affecting the state design and resulting in invalid training. Therefore, the state had to be defined from a single batch of sampling data. During DQN modeling, the value of the measurement error was normalized; the displacement features were converted to facilitate observation and training. Therefore, the desired command and actual compensated error values were used as the input state for the DQN model.

In this research, consecutive DQN states comprised the cumulative offset error data of 10 time steps of the XXY platform. This method enabled increasing the state data set by selecting different 10-step slices of the data set; for example, a second data set could be produced by selecting the state one time step after the initial state and all subsequent states separated from it by a multiple of 10 time steps. This method produced 10 data sets for each run. Moreover, the DQN can move forward or backward in time between states, thereby offering further opportunities for learning. The aforementioned method substantially improved the training efficiency.

### 4.3. Action Design of the DQN Model

The action is an element of the RL model. To enable effective interactions with the environment and learning, the action space was established and provided to the agent for decision-making. The platform motion actions are the Up, Down, and Hold commands. Up and Down are defined as displacement compensation in the same and opposite direction, respectively, as the platform motion, and Hold halts the platform. Table 2 presents the agent’s action set.

### 4.4. Reward Design of the DQN Model

**Figure 8:**
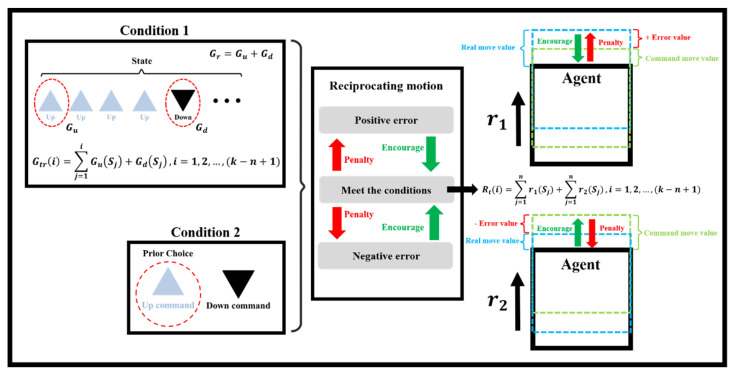
Reward and penalty rules for platform positioning motions.

In RL, the agent must interact with the environment by selecting actions to obtain rewards. Through an effective reward design, the system can establish criteria for judging decision quality. The reward may be positive or negative. The reward method should be in accordance with characteristics of the moving-platform, ball-screw, feed-drive system. A diagram of the reward rules selected in this study is presented in Figure 8. The right side of Figure 8 indicates rewards for the positioning motion of the platform (dashed green line). The model was rewarded (encouraged, green arrow symbol) if it commanded backward or forward displacement for an overshooting or undershooting command, respectively (dashed cyan line); the model was penalized if it took the opposite action. Equation (10) defines the total reward value *R_t_*.

$$r_{1}≜Reward\;for\;\left[real\;move-command\;move\right]$$

$$r_{2}≜Reward\;for\;\left[-\left(real\;move-command\;move\right)\right]$$

(10) $$R_{t}\left(i\right)=\overset{n}{\underset{j=1}{\sum }}r_{1}\left(S_{j}\right)+\overset{n}{\underset{j=1}{\sum }}r_{2}\left(S_{j}\right),i=1,2,\ldots ,\left(k-n+1\right)$$

where *S* indicates the state of motion, *k* is the total number of positioning motions, and *n* is the number of motions in the current state.

When the ball-screw drive system was frequently moved back and forth, heat was produced and wear occurred, which resulted in ball-screw elongation, reduced stiffness, and increased positioning error. Moreover, positioning error would have accumulated because of backlash if numerous error alternating compensation commands were requested. Therefore, a reward was given if the first command was Up (top left of Figure 8), that is, the follow-up motions were in the direction of the platform motion. This rule reduced the frequency of the feed-drive motor reversing its direction. Heuristically, this phenomenon causes reductions in temperature increases and frictional wear. The first Down command (top left of Figure 8) was also rewarded. Therefore, the transform reward function of *G_tr_* for condition 1 is defined as in Equation (11).

$$G_{r}=G_{u}+G_{d}$$

(11) $$G_{tr}\left(i\right)=\overset{i}{\underset{j=1}{\sum }}G_{u}\left(S_{j}\right)+G_{d}\left(S_{j}\right),i=1,2,\ldots ,\left(k-n+1\right)$$

where *S* indicates a change in platform direction, and the subscripts *u* and *d* indicate the first Up and Down commands, respectively. However, the agent can still randomly search for an improved policy by reversing the error compensation direction. For example, if error compensation requires movement in the same direction in consecutive steps, this causes more time steps based on the feedback control policy. When the hold command is given, many back-and-forth motions might occur for a steady-state compensation policy based on previous errors. Moreover, a greedy agent might always select the reverse action of the prior action. This scenario is similar to that of a feedforward controller and nonminimum phase control behavior. Therefore, the error compensation should minimize back-and-forth ball-screw rotations. Consequently, the occurrence of some backward motions followed by a forward compensation is in accordance with the RL policy, as indicated by condition 2 in the bottom left of Figure 8. Therefore, the total reward contains the policies of condition 1 (Equation (11)) and condition 2.

### 4.5. Neural Network Design of the DQN Model

Initially, the traditional Q-learning table method was considered for the proposed architecture; however, the prediction results for different positioning actions were poor. Therefore, the DQN model extended by Q-learning was selected. Compared with traditional Q-learning, in which the Q value is searched for and iterated one step at a time, the DQN uses a neural network to perform search and iteration and directly outputs the Q value.

**Figure 9:**
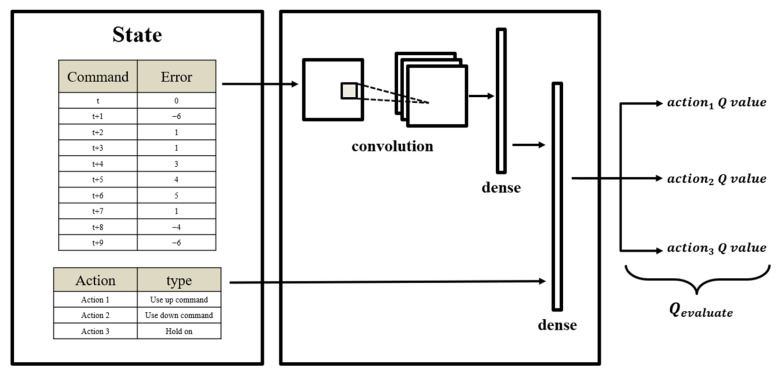
Deep Q-learning network (DQN) architecture for the XXY platform.

The DQN architecture used in the research is shown in Figure 9. The input of the network is the error data set of the XXY platform and the action space data set. The XXY platform error data set was passed through a convolutional neural network layer and combined with the action space data set. The layer denoted as “dense” in Figure 9 is the final fully connected layer, which outputs a set of Q values.

The parameters used to train the DQN agent are listed in Table 3. The initial value is the agent training capacity, which strongly affects the agent’s ability to recognize the environment and its sensitivity to environmental factors. This value was preset to 1000 in this study, and the storage memory space for the replay experience size was set as 3000. A total of 12 experienced replay memories were added to the training data set. The linear annealing epsilon-greedy parameter ε was set to decrease linearly from 0.8 to 0.01 as training continued. The learning rate was 0.9, with the discount factor being 0.95. The state size was set as 10, and the data mode was selected as an interval of 1.

## 5. Simulation and Experimental Results for the Model-Free DQN Model

**Figure 10:**
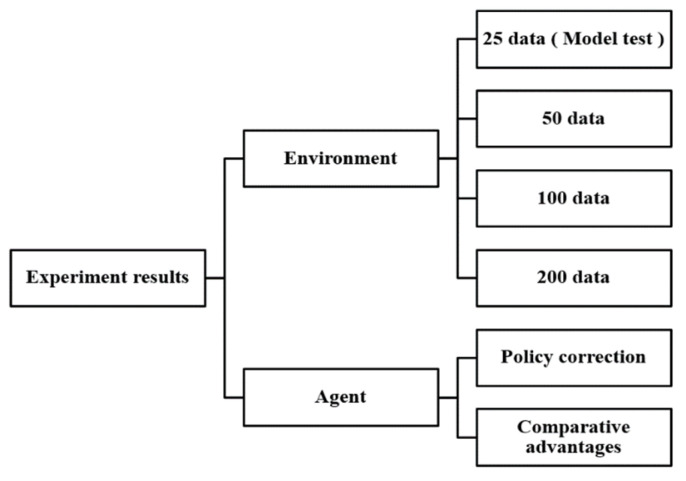
Overview of experiments with various quantities of positioning error data and different agent behaviors and policies.

The input data for environmental interactions are listed in Figure 10. Initially, 25 data were input, and this number was increased to 50, 100, and 200 for further evaluations of the agent’s performance, which was determined as the designated total reward and total transform reward policies in Equations (10) and (11).

### 5.1. DQN Training

In the experiment, only 25 XXY platform positioning errors were initially used to establish the RL environment. The effectiveness of the agent was evaluated for this small state space by comparing the predicted DQN with the target DQN by determining the trend of the loss function. The predicted Q value approached the target Q value as the loss function converged; thus, the reward and loss functions, the DQN parameters, and the action space were unchanged.

The loss function was intended to cause the current Q value of the neural network to approach the target Q value in the DQN model through direct environmental interactions. In DQN iterations (epochs and episodes), if the loss function is small, the change in the Q value is also small. In this case, the predicted reward is similar to the actual reward obtained through environmental interactions; thus, the Q value output by the neural network approximates the Q value output by the target network. The simulation results validated the DQN output.

**Figure 11:**
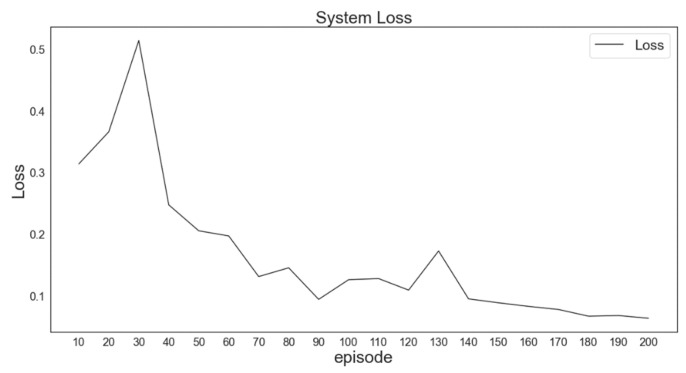
Loss function after training with 25 data for 200 generations.

By observing the convergence of the loss function, the situation of the agent in model training could be inferred. Moreover, whether the DQN model had completed training could be determined. In Figure 11, the loss function, namely the mean absolute error of the XXY platform positioned on one axis, becomes sufficiently small after 200 generations of training despite a spike within training epochs 20–40. *This apparent divergence followed by convergence is attributable to the greedy parameter (ε-greedy) in the early stage of training, which increases the probability that the agent performs random actions for exploration. The loss function gradually converges as the agent begins to understand the environment and learns the hidden rules of the XXY platform system.*

### 5.2. Experimental Validation of the Results of the DQN Model

**Figure 12:**
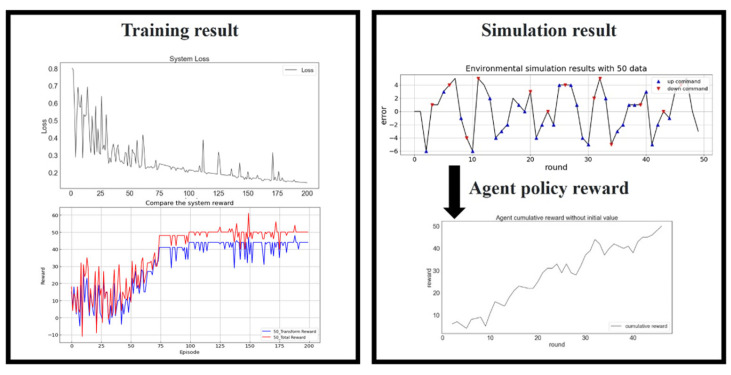
Training results for 50 data. Loss function (**top left**), reward (**bottom left**), simulated platform positioning (**top right**), and the agent policy reward (**bottom right**).

Figure 12 presents the training results for 50 data. The loss function, total reward, and total transform reward validated the training results. The loss function (top left of Figure 12) converged after 200 training generations. The simulation results (top right of the Figure 12) indicated that the agent could effectively control the platform with Up and Down commands by compensating for the positioning error for 50 iterations. The increasing policy reward (bottom right of Figure 12) revealed that the agent became an excellent positioning compensation proxy. The agent policy reward increased as the number of iterations increased. The simulation results revealed that the control process was partly random but partly influenced by the agent, as indicated by the plot of the transform reward in the bottom left of Figure 12.

**Figure 13:**
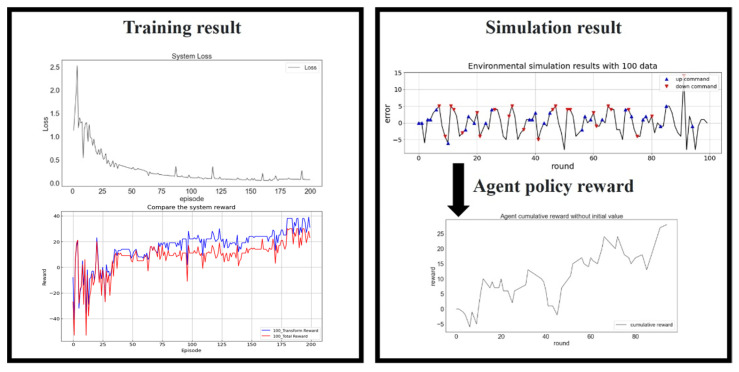
Training results for 100 data. Loss function (**top left**), reward (**bottom left**), simulated platform positioning (**top right**), and the agent policy reward (**bottom right**).

Figure 13 reveals that the loss function still converged after 200 episodes for 100 training data. A comparison of the top left and bottom left of Figure 13 revealed that the agent performed exploration to identify a better reward policy until episode 35. The policy was then continually modified in each iteration. Although some spikes appeared in the loss function during RL, the bottom left of Figure 13 reveals that these spikes corresponded to reductions in the reward.

**Figure 14:**
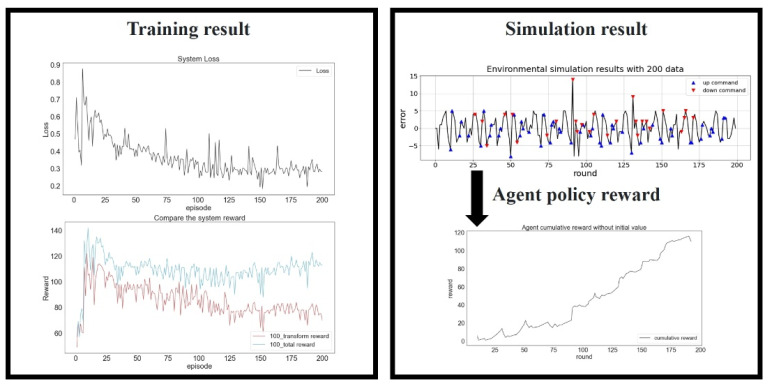
Training results for 200 data. Loss function (**top left**), reward (**bottom left**), simulated platform positioning (**top right**), and the agent policy reward (**bottom right**).

In the final experiment, 200 data were input as the training data (Figure 14). The training difficulty was the highest in this environment, and the initial random strategy provided poor results. However, the loss function decreased and converged as the number of training generations increased. Although slight fluctuations occurred in the loss function plot, the higher numbers of state transition and decision-making possibilities finally resulted in the highest total reward.

## 6. Conclusions

In this study, an in-lab CCD visual image recognition system was used to observe the displacement of an XXY platform. The actual position of the platform was obtained using a dial indicator in the offline mode to provide more accurate positioning information to the control system. The offset error was calculated from a visual servo image, and the actual positions were used as the input data set for a DQN RL model with an action data set of platform commands to improve platform positioning. Time-series prediction and the Markov stochastic control process of the DQN were used to compensate for the displacement deviations when the XXY platform was actuated. The error compensation results from the two reward policies verified that the developed model could effectively control the XXY platform. Thus, this research provides a novel movement compensation method for improving positioning control by deploying a DQN. The simulation results and methodology of this study can be applied in feedback control applications.

## Figures and Tables

**Table 1:** Positioning data set for XXY platform errors.

Position	Actual Move (μm)	Error (μm)
0~100	100	0
100~200	94	−6
200~300	101	1
300~400	101	1
…	…	…
100~0	−97	3

**Table 2:** Agent action set.

Action Scenario	Perform
Action 1	Use Up command
Action 2	Use Down command
Action 3	Hold on

**Table 3:** Parameters of the DQN.

Parameter	Value
Initial Value	1000
Memory Size	3000
Batch Size	12
ε−greedy_start	0.8
ε−greedy_min	0.01
ε−decade rate	0.999
Learning Rate α	0.9
Discount Factor η (Gamma)	0.95
State_size	10
Skip	1

## Data Availability

Not applicable.
